# *smu_1558c*-mediated regulation of growth and biofilm formation in *Streptococcus mutans*

**DOI:** 10.3389/fmicb.2024.1507928

**Published:** 2025-01-17

**Authors:** Jing Zhou, Qizhao Ma, Jingou Liang, Yangyang Pan, Yang Chen, Shuxing Yu, Yaqi Liu, Qiong Zhang, Yuqing Li, Jing Zou

**Affiliations:** ^1^State Key Laboratory of Oral Diseases, National Clinical Research Center for Oral Diseases, West China Hospital of Stomatology, Sichuan University, Chengdu, China; ^2^Department of Pediatric Dentistry, West China Hospital of Stomatology, Sichuan University, Chengdu, China; ^3^Department of Pediatric Dentistry, School and Hospital of Stomatology, Wenzhou Medical University, Wenzhou, China; ^4^Department of Pediatric Dentistry, Peking University School and Hospital of Stomatology, Beijing, China

**Keywords:** dental caries, *Streptococcus mutans*, acetyltransferase, biofilms, water-insoluble extracellular polysaccharides, ClpL

## Abstract

*Streptococcus mutans* is a key etiological agent in dental caries, owing to its strong ability to form biofilms through carbohydrate fermentation. Protein acetylation, facilitated by GNAT family acetyltransferases, plays a critical regulatory role in bacterial physiology, but its impact on *S. mutans* remains largely unexplored. In this study, we investigated the role of the GNAT family acetyltransferase encoded by *smu_1558c* in regulating the growth and biofilm formation of *S. mutans*. The deletion of *smu_1558c* resulted in impaired growth, reduced biofilm formation, and diminished synthesis of water-insoluble extracellular polysaccharides (EPS). Proteomic analysis revealed 166 differentially expressed proteins in the deletion mutant, with significant enrichment in pathways related to carbohydrate transport and metabolism, and translation. Notably, glucosyltransferases GtfB and GtfC, key enzymes in biofilm formation, were significantly downregulated in the deletion mutant, while ClpL, a Clp-like ATP-dependent protease involved in protein homeostasis under stress conditions, was highly upregulated. These findings highlight that acetyltransferase *smu_1558c* plays a crucial role in the growth, biofilm formation, and EPS synthesis of *S. mutans* through its regulation of carbohydrate transport and metabolism pathways, as well as stress response mechanisms. This study provides novel insights into the molecular mechanisms governing *S. mutans* pathogenicity and suggests potential therapeutic targets for caries prevention.

## 1 Introduction

Dental caries is one of the most prevalent infectious diseases worldwide, imposing significant economic burdens and affecting the quality of life for individuals (Pitts et al., 2017; Simón-Soro and Mira, 2015). The disease is driven primarily by the dysbiosis of the dental plaque microbiome, characterized by an overgrowth of cariogenic microorganisms (Rosier et al., 2018; Lamont et al., 2018). Among these, *Streptococcus mutans* is recognized as a primary causative agent due to its physiological traits of high acidogenicity, aciduricity, and the ability to produce exopolysaccharides (Cugini et al., 2019). In the presence of sucrose, *S. mutans*-derived glucosyltransferases (Gtfs) synthesize polysaccharides that form an extracellular polymeric matrix (Zhang et al., 2021). This matrix enhances the local accumulation and cohesion of microbes on teeth surfaces, providing a protective environment that enhances the bacteria’s tolerance to environmental stresses (Nishimura et al., 2012). Over time, *S. mutans* and other embedded acidogenic organisms ferment sugars within this matrix, creating a highly acidic microenvironment that ultimately leads to the demineralization of tooth enamel and dentin, resulting in tooth decay (Klein et al., 2015; Koo et al., 2013). The pathogenicity of *S. mutans* is thus intricately linked to its biofilm formation and metabolic activities.

Protein acetylation is a crucial post-translational modification (PTM) that modulates various protein functions, playing a significant role in cellular processes (Narita et al., 2019; Shvedunova and Akhtar, 2022). In prokaryotes, protein acetylation is primarily facilitated by enzymes from the GCN5-related *N*-acetyltransferases (GNATs) family (Carabetta and Cristea, 2017; Ren et al., 2017). These enzymes transfer acetyl groups from donor molecules, such as acetyl coenzyme A (acetyl-CoA), to lysine residues on target proteins, thereby influencing their activity, stability, and interactions. GNAT family acetyltransferases are involved in diverse biological processes, including bacterial drug resistance, transcription regulation, oxidative stress response, heat resistance, and metabolism (VanDrisse and Escalante-Semerena, 2019; Christensen et al., 2019; Liu et al., 2021). This widespread involvement underscores the importance of GNAT family acetyltransferases in bacterial adaptability and survival (Ma et al., 2021; Burckhardt and Escalante-Semerena, 2020).

Based on NCBI genome annotation, we identified 15 GNAT family acetyltransferases in *S. mutans* (Ajdic et al., 2002). In our previous research, we constructed overexpression strains for each of these acetyltransferases to investigate their functions. Our findings revealed that ActA and ActG significantly influence the acid production and biofilm formation capabilities of *S. mutans*, respectively (Ma et al., 2022; Ma et al., 2021). However, the functions of the remaining acetyltransferases are not well understood. To further elucidate their roles, we also constructed deletion mutants for each acetyltransferase, allowing us to study the phenotypic consequences of their absence. These investigations aim to uncover the specific contributions of each acetyltransferase to the physiological and pathogenic traits of *S. mutans*.

In this study, we focused on *smu_1558c*, a putative GNAT family acetyltransferase, to elucidate its role in the growth and biofilm formation of *S. mutans*. By constructing a mutagenesis library of GNAT family acetyltransferases, we identified that the deletion of *smu_1558c* significantly impaired bacterial growth. We further investigated the phenotypic consequences of *smu_1558c* deletion on biofilm formation and EPS synthesis. In addition, we used quantitative proteomics to explore the global changes in protein expression associated with the deletion of *smu_1558c*, highlighting significant alterations in pathways related to carbohydrate transport and metabolism, and stress response mechanism. These results provide new insights into the regulatory mechanisms governing *S. mutans* biofilm formation and pathogenicity.

## 2 Materials and methods

### 2.1 Bacteria strains and growth conditions

*S. mutans* UA159 was obtained from the American Type Culture Collection (ATCC 700610). *S. mutans* and its derivatives were routinely grown in a brain–heart infusion broth (BHI, Difco, Sparks, MD, United States) at 37°C anaerobically (90% N_2_, 5% CO_2_, 5%H_2_). For biofilm formation assays, additional sucrose (1%, w/v) was supplied to BHI broth. *Escherichia coli* (*E. coli*) strains were grown in Luria–Bertani medium (LB; Difco, Sparks, MD, United States) supplemented with spectinomycin (100 μg/mL) when necessary. Bacterial strains, plasmids, and primers used in our study are listed in Supplementary Table S1.

### 2.2 Construction of deletion mutants

The in-frame deletion mutants of *S. mutans* UA159 were constructed using a two-step transformation method as previously described (Xie et al., 2011). In brief, approximately 1 kb regions upstream and downstream of the open reading frame of the target genes were amplified using primers upF/upR and dnF/dnR, respectively. These PCR products were then ligated into the IFDC2 by overlapping PCR with upF/dnR primers for each gene. The resultant fragment was transformed into UA159, and transformants were selected on BHI plates containing erythromycin (12.5 μg/mL). For the markerless in-frame deletion of *smu_1558c*, upstream and downstream fragments of the targeted genes were amplified using primers *smu_1558c* upF/*smu_1558c* updnR and *smu_1558c* dnF/*smu_1558c* dnR, respectively, and ligated using primers *smu_1558c* upF/*smu_1558c* dnR. The resulting fragments were transformed into the first-step selected strains, which were then selected on BHI plates with *p*-chlorophenylalanine (4 mg/mL). Deletion mutants were confirmed by PCR and sequencing.

### 2.3 Construction of overexpression strains

To construct the overexpression strains of *smu_1558c*, the *smu_1558c* and *ldh* promoter were amplified from *S. mutans* genomic DNA by PCR using primers *smu_1558c*-F*/ smu_1558c*-R-*Sal*I and *ldh*-F-*Sac*I*/ldh*-R, respectively. These fragments were assembled by overlap extension PCR with primers *ldh*-F-*Sac*I/*smu_1558c*-R-*Sal*I. The *E. coli*-*Streptococcus* shuttle vector pDL278 (LeBlanc et al., 1992) was digested with *Sac*I and *Sal*I and ligated with the purified PCR product using T4 ligase (Takara Bio, Japan) to yield pDL278-*smu_1558c*. The recombined plasmids were transformed into the UA159 and confirmed by PCR and sequencing.

### 2.4 Growth curve measurement

Overnight cultures of *S. mutans* and its derivatives were subcultured in BHI broth until the optical density at 600 nm (OD_600 nm_) to 0.5 and then diluted 1:100 in fresh BHI broth. Bacterial growth was monitored hourly by measuring OD_600 nm_ using a microplate reader (BioTek, United States). Each strain was performed in triplicate, with the experiment being independently repeated three times.

### 2.5 Biofilm formation assay and quantification of water-insoluble EPS

Overnight cultures of *S. mutans* and its derivatives were grown in sucrose-supplemented BHI broth in 48-well polystyrene plates for 6 h and 24 h. Each strain was performed in triplicate, with the experiment being independently repeated three times. Biomass was quantified using the crystal violet staining method as previously described (Gong et al., 2021; Xu et al., 2011). In brief, the medium was removed, and the biofilm was washed twice with phosphate-buffered saline (PBS) solution and then stained with 0.1% crystal violet for 15 min. The dye was dissolved in 33% acetic acid, and the absorbance was measured at OD_600 nm_ (BioTek, United States).

The water-insoluble extracellular polysaccharides (EPS) in biofilms were quantified according to the previously described method with minor modifications (Xiang et al., 2019; Chen et al., 2021). Biofilms were incubated for 6 h and 24 h in 48-well polystyrene plates. Each strain was performed in triplicate, with the experiment being independently repeated three times. Cells in each well were resuspended in 1 mL of PBS and centrifuged at 10,000 g for 10 min at 4°C. The supernatant was discarded, and the pellet was washed twice with PBS. The pellet was then resuspended in 1 mL of 1.0 M NaOH and incubated for 2 h at 37°C to extract the water-insoluble glucans. The alkali-soluble carbohydrate solution (200 μL) was mixed with anthrone-sulfuric acid reagent and heated at 95°C for 5 min. The absorbance was measured at OD_625 nm_ (BioTek).

### 2.6 Scanning electron microscopy analysis

Overnight cultures of *S. mutans* and its derivatives were diluted 1:10 into fresh BHI broth and grown to an OD_600 nm_ of 0.5 and then diluted 1:100 in BHI broth supplemented with 1% sucrose (w/v) at 37°C under anaerobic conditions on glass coverslips in 12-well plates for 6 h and 24 h. After biofilm formation, the biofilms were fixed with 2.5% paraformaldehyde, dehydrated with a gradient series of ethanol, dried, and gold-coated. The samples were then observed under SEM at 1,000×, 5,000×, and 20,000× magnification.

### 2.7 Confocal laser scanning microscopy analysis

The bacterial cells and EPS of *S. mutans* biofilm were labeled with SYTO 9 (Molecular Probes, Invitrogen, United States) and Alexa Fluor 647-labeled dextran conjugate (Molecular Probes, Invitrogen, United States), respectively, as previously described (Xiao et al., 2012; Yu et al., 2024). The cells were grown on a *μ*-Slide 8 Well culture plate (ibidi GmbH, Germany) for 24 h. Imaging was performed using CLSM (Nikon A1, Japan) with a 60× oil immersion lens. We collected at least three random fields of view for each strain and conducted three independent biological replicates. Fluorescence was detected at 495–515 nm for SYTO 9 and 655–690 nm for Alexa Fluor 647 with the same settings for each time-point biofilm. Quantified analysis was by Image J. Image files were converted to individual grayscale multipage Tiff images for each slice. Grayscale images were converted into bio-formats and consistent thresholding parameters for all images. Biomass of bacteria and EPS were calculated by COMSTAT 2.1 plug-in component individually (Tian et al., 2010).

### 2.8 SDS-PAGE analysis

Overnight cultures of *S. mutans* and its derivatives were diluted 1:10 into fresh BHI broth and grown to an OD_600 nm_ of 0.5. The cells were harvested, homogenized, and centrifuged, and the proteins in the supernatant were quantified using a bicinchoninic acid (BCA) assay. Protein samples were mixed with 5× loading buffer and separated by electrophoresis on 8–20% Precast-Gel (Solarbio, China).

### 2.9 Proteome analysis

For proteome analysis, *S. mutans* UA159 and UA159 Δ*1558c* were cultured overnight, diluted 1:10 into BHI broth, and grown to an OD_600 nm_ of 0.5. Then, cells were collected by centrifuge at 4,000 g for 10 min at 4°C and snap-frozen in liquid nitrogen for 15 min. Subsequently, the samples were sent for Tandem Mass Tag (TMT)-based quantitative proteomics (Majorbio Co., Ltd., China). All analyses were performed on the free online Majorbio Cloud Platform.^1^ Gene-annotation enrichment analysis and functional annotation clustering of the differentially expressed proteins (DEPs) were performed using the DAVID bioinformatics resources V6.7.^2^ Significant enrichment categories with a *p-*value less than 0.05 were considered. Protein–protein interactions (PPIs) for DEPs were analyzed using Cytoscape V3.9.1 (Institute of Systems Biology, Seattle, WA). The PPI network was obtained from the STRING database.^3^

### 2.10 Statistical analysis

Statistical analyses were conducted using Prism 8 (Graph Pad, United States) and SPSS software V25.0 (SPSS Inc., America). Parametric data are presented as means, with error bars representing standard deviations. An unpaired Student’s *t*-test was used to compare means between two groups, while one-way analysis of variance (ANOVA) followed by the Student–Newman–Keuls test was used to compare means among multiple groups. For experiments involving two factors, two-way analysis of variance (ANOVA) was performed, followed by Sidak’s *post-hoc* test for pairwise comparisons. Statistical significance was accepted at a two-tailed *p*-value of < 0.05.

## 3 Results

### 3.1 Deletion of *smu_1558c* impaired the growth of *Streptococcus mutans*

To investigate the role of acetyltransferases in the growth of *S. mutans*, we constructed a mutagenesis library of GNAT family acetyltransferases and measured the growth differences between the mutant strains. The initial screen revealed that the UA159 Δ*1558c*::IFDC2 strain exhibited delayed growth during the logarithmic phase compared to the parental strain UA159 (Supplementary Figure S1). To further elucidate the impact of *smu*_*1558c* on the growth of *S. mutans*, the markerless in-frame deletion strain UA159 Δ*1558c* and the overexpression strain UA159/pDL278-*1558c* were constructed.

**Figure 1:**
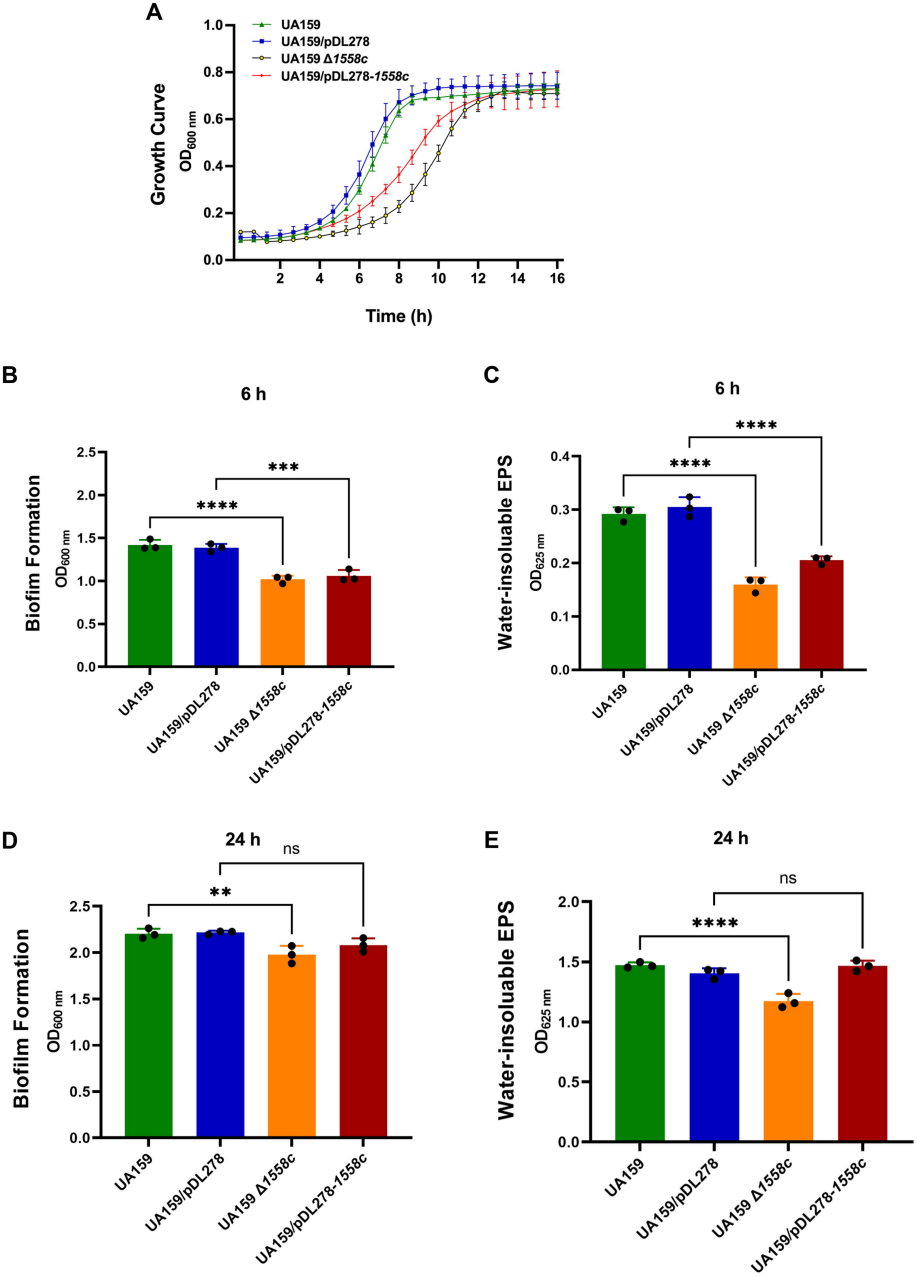
Effect of *smu_1558c* on bacterial growth, biofilm formation, and water-insoluble EPS synthesis in *S. mutans*. **(A)** Growth curves of UA159, UA159/pDL278, UA159 Δ*1558c*, and UA159/pDL278-*1558c* in anaerobic conditions for 16 h. **(B–E)** The biofilm biomass was determined by crystal violet staining assay and anthrone-sulfuric acid method when cultured in BHIS (1% sucrose, wt/vol) in anaerobic conditions for 6-h **(B,C)** and 24-h **(D,E)**, respectively. Data represent three independent experiments (** *p* < 0.01, *** *p* < 0.001, or **** *p* < 0.0001; ns, not significant).

Consistently, the markerless in-frame deletion strain UA159 Δ*1558c* was significantly slower during the logarithmic phase compared to the wild-type strain UA159 (Figure 1A). Similarly, the overexpression strain UA159/pDL278-*1558c* also exhibited a delayed growth rate during the logarithmic phase compared to the control strain UA159/pDL278, although this delay was less pronounced than that observed in the UA159 Δ*1558c* strain (Figure 1A). However, no significant differences were observed among all strains during the stationary phase. These results suggest that the *smu_1558c* plays a critical role in regulating the growth of *S. mutans*.

### 3.2 Deletion of *smu_1558c* inhibited the water-insoluble EPS synthesis and biofilm formation

To better understand the role of *smu_1558c* in the pathogenesis of *S. mutans*, we evaluated its impact on biofilm formation and water-insoluble EPS synthesis, the primary virulence factors in the development of dental caries. Biofilm biomass and water-insoluble EPS synthesis were quantified using crystal violet dye staining and the anthrone-sulfuric acid method, respectively. As shown in Figures 1B,C, the deletion strain UA159 Δ*1558c* exhibited a significant reduction in biofilm biomass and water-insoluble EPS production in 6-h biofilms, compared to the wild-type strain UA159. Similarly, the overexpression strain UA159/pDL278-*1558c* also showed a significant reduction in biofilm biomass and water-insoluble EPS production in 6-h biofilms compared to the control strain UA159/pDL278, though the reduction in water-insoluble EPS production was less pronounced than that observed in the *smu_1558c* deletion strain. For 24-h biofilms, the deletion strain UA159 Δ*1558c* continued to show a significant reduction in biofilm biomass and water-insoluble EPS production compared to the wild-type strain UA159 (Figures 1D,E). However, no significant differences in biofilm biomass and water-insoluble EPS production between overexpression strain UA159/pDL278-*1558c* and its control strain UA159/pDL278 were observed (Figures 1D,E).

### 3.3 Deletion of *smu_1558c* altered the three-dimensional structure of biofilms

**Figure 2:**
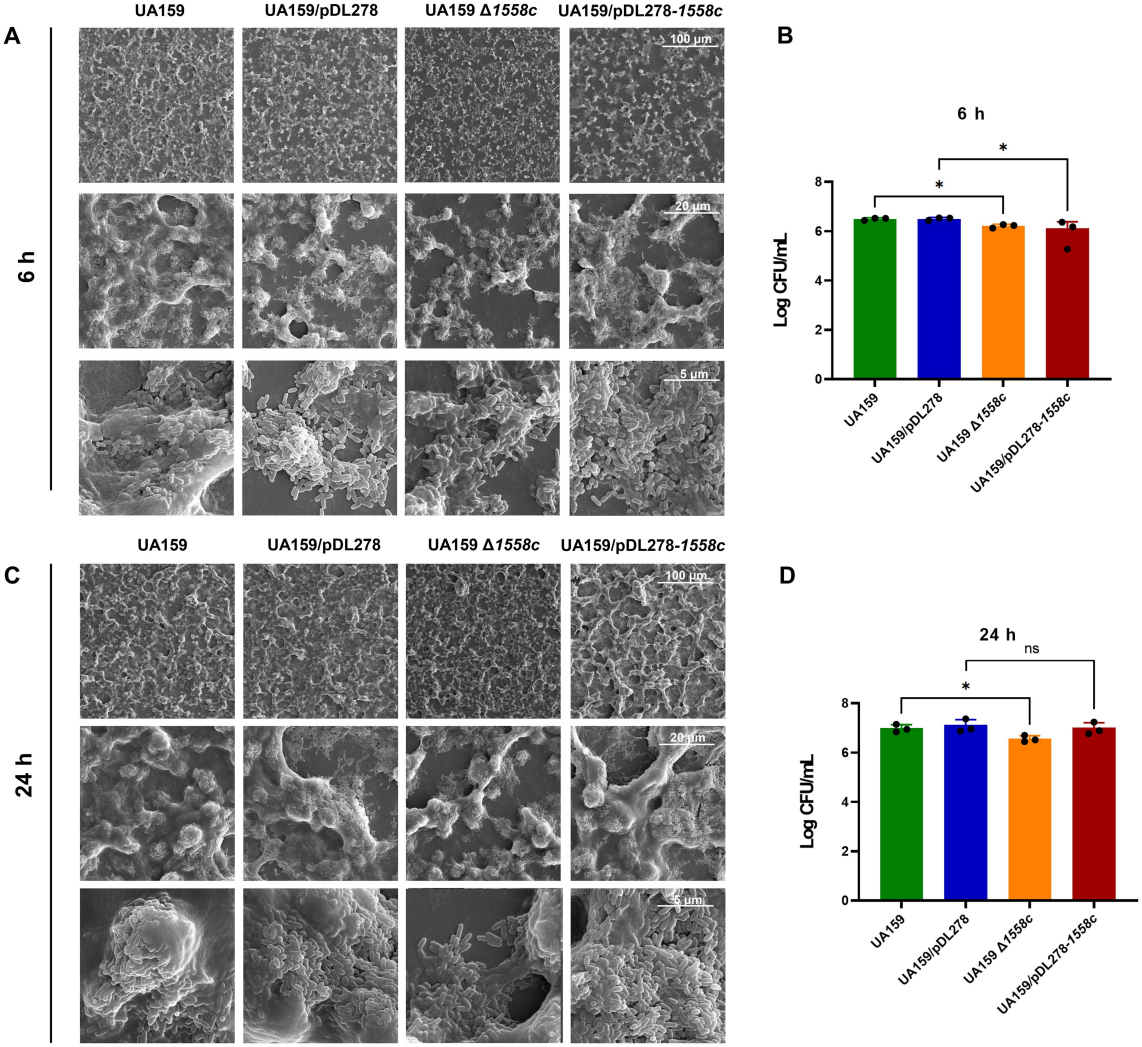
Effect of *smu_1558c* on biofilm architecture of *S. mutans*. **(A,C)** Scanning electron microscopy analysis of the structure of 6-h **(A)** and 24-h **(C)** biofilms. Images were captured at 1,000×, 5,000×, and 20,000× magnification. Representative images are shown from at least 5 randomly selected positions of each sample. **(B,D)** The colony-forming units (CFUs) from 6 h **(B)** and 24 h **(D)** biofilms were counted. The results are presented as mean ± SD. Data represent three independent experiments (* *p* < 0.05; ns, not significant).

To further investigate the structural changes caused by the deletion of *smu_1558c*, we used scanning electron microscopy (SEM) to analyze 6-h and 24-h biofilms. As shown in Figure 2A, the deletion strain UA159 Δ*1558c* exhibited a porous and less compact biofilm structure compared to the wild-type strain UA159 in the 6-h biofilms. This spongy structure with reduced water-insoluble EPS is consistent with the decreased biofilm biomass and EPS synthesis observed in Figures 1B,C. The overexpression strain UA159/pDL278-*1558c* also showed a similar, though slightly less pronounced, porous biofilm structure compared to the control strain UA159/pDL278. For 24-h biofilms, the deletion strain UA159 Δ*1558c* continued to exhibit a relatively porous structure with reduced biofilm density compared to the wild-type strain UA159, whereas the overexpression strain UA159/pDL278-*1558c* did not show significant structural differences from its control strain UA159/pDL278 (Figure 2C). To correlate these structural changes with bacterial viability, we measured colony-forming units (CFUs) from 6-h and 24-h biofilms (Figures 2B,D). In the 6-h biofilms, the deletion strain UA159 Δ*1558c* and the overexpression strain UA159/pDL278-*1558c* showed a significant reduction in CFUs compared to UA159 and UA159/pDL278, indicating that the reduced biofilm biomass may be partially attributed to lower bacterial cell numbers (Figure 2B). In the 24-h biofilms, the deletion strain UA159 Δ*1558c* continued to exhibit a significant reduction in CFUs compared to UA159, while no significant differences were observed between UA159/pDL278-*1558c* and UA159/pDL278 (Figure 2D).

**Figure 3:**
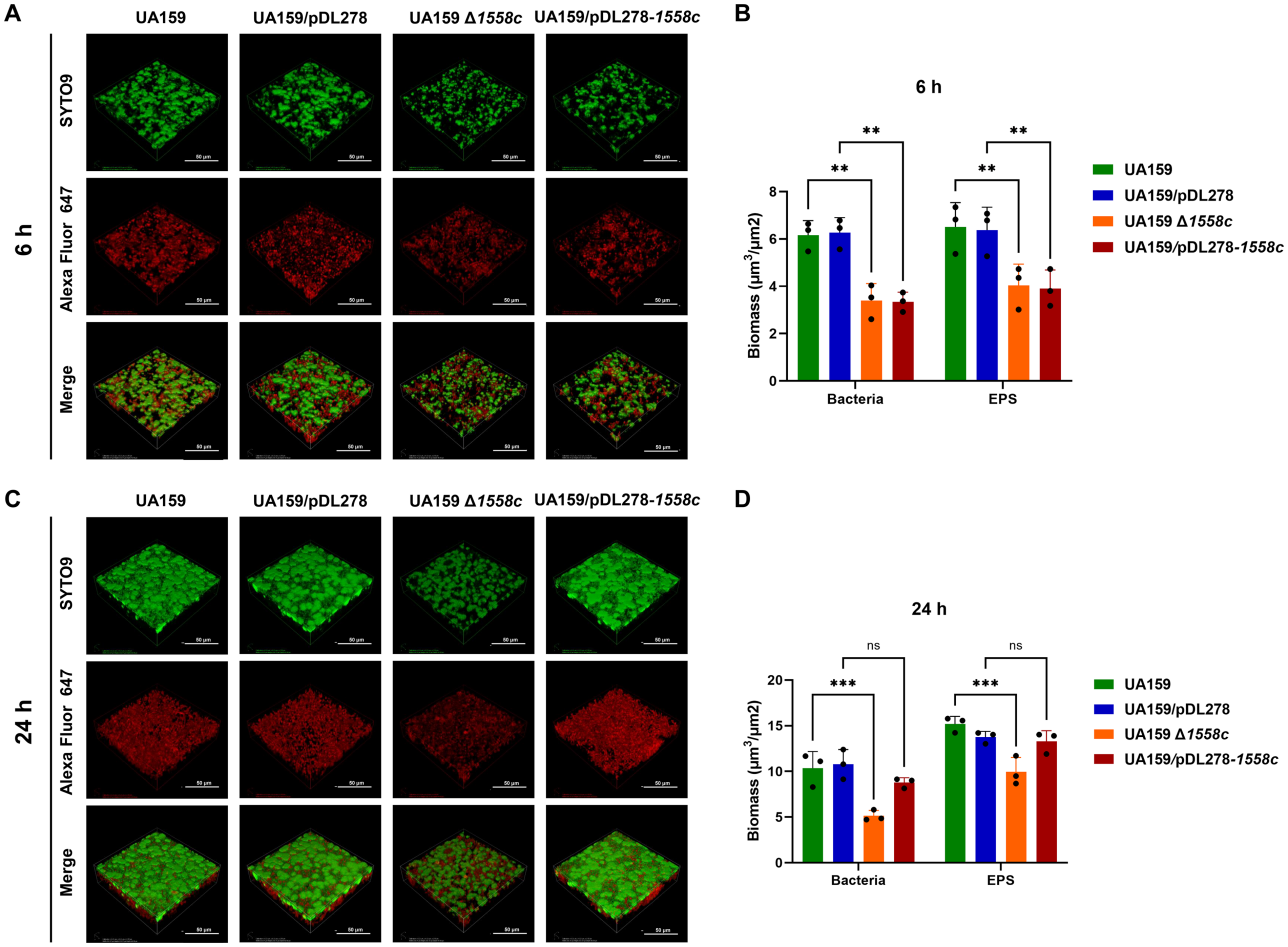
Effect of *smu_1558c* on biofilm architecture and composition of *S. mutans*. **(A)** Confocal laser scanning microscopy (CLSM) images of three-dimensional visualization of bacteria (green) and EPS (red) of 6-h biofilms. **(B)** Biomass analysis of 6-h biofilms. **(C)** CLSM images of three-dimensional visualization of bacteria (green) and EPS (red) of 24-h biofilms. **(D)** Biomass analysis of 24-h biofilms. Images were captured at 60× magnification, and the three-dimensional reconstructions of biofilms were performed. Data represent three independent experiments (** *p* < 0.01, *** *p* < 0.001; ns, not significant).

In addition, we also observed the biofilm structure and water-insoluble EPS using confocal laser scanning microscopy (CLSM). Bacterial cells were stained with SYTO 9 (green), while EPS were labeled with Alexa Fluor 647 (red). In the 6-h biofilms, UA159 Δ*1558c* and UA159/pDL278-*1558c* exhibited reduced bacterial density and decreased EPS production compared to UA159 and UA159/pDL278, respectively (Figure 3A). The CLSM analysis revealed that the deletion strain UA159 Δ*1558c* exhibited a significant reduction in biofilm biomass compared to the wild-type strain UA159 (Figure 3B). In the 24-h biofilms, UA159 Δ*1558c* continued to show sparse biofilm and minimal EPS compared to UA159 and no significant differences between UA159/pDL278-*1558c* and UA159/pDL278 (Figure 3C). The CLSM analysis showed that the UA159 Δ*1558c* strain continued to exhibit a significant reduction in biofilm biomass compared to the wild-type strain UA159. However, no significant differences in biofilm biomass were observed between the overexpression strain UA159/pDL278-*1558c* and its control strain UA159/pDL278 (Figure 3D). These results indicate that the deletion of *smu*_*1558c* impacts the growth, biofilm formation, and EPS synthesis in *S. mutans*, leading to structural changes in the biofilm and affecting its density and composition.

### 3.4 Quantitative proteomics analysis of the UA159 Δ*1558c*

**Figure 4:**
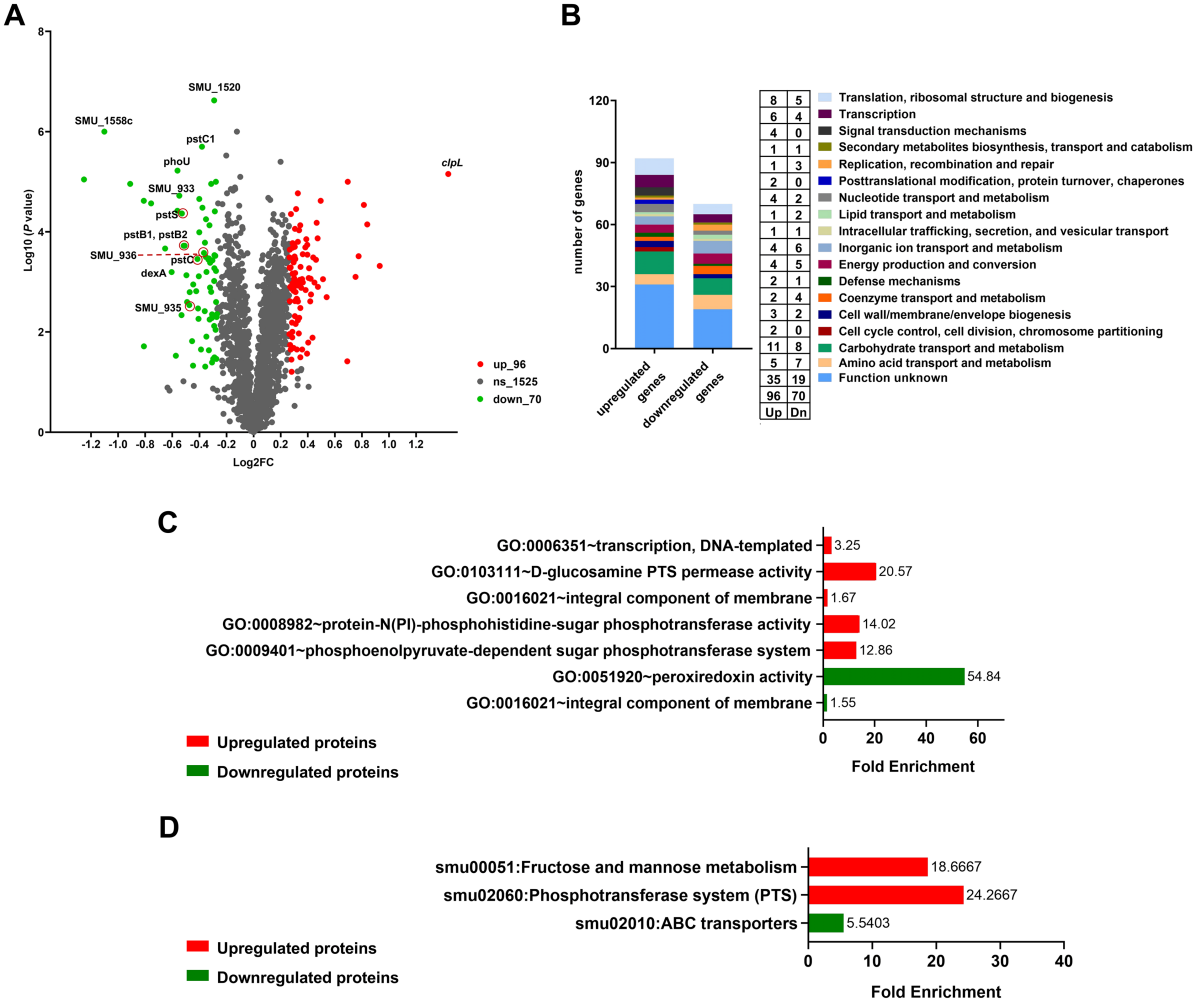
Differential proteomic analysis of UA159 and UA159 Δ*1558c*. **(A)** Volcano plot shows the differentially expressed proteins in the deletion strain UA159 Δ*1558c* compared to the wild-type strain UA159. Proteins significantly upregulated (up) are shown in red, while downregulated proteins (dn) are in green. Non-significant proteins (ns) are shown in gray. **(B)** Functional categorization of differentially expressed proteins based on clusters of orthologous genes (GOG) classification. **(C,D)** GO enrichment analysis **(C)** and KEGG pathway enrichment analysis **(D)** of the upregulated and downregulated proteins in the UA159 Δ*1558c*.

To further investigate the regulatory mechanisms underlying the observed phenotypic changes, we examined the protein profiles of cell lysates from the UA159, UA159/pDL278, UA159 Δ*1558c*, and UA159/pDL278-*1558c* strains using SDS-PAGE analysis. Although the banding patterns of most proteins were similar across all strains, there was a significantly upregulated protein band in the molecular weight range of 70 kDa to 100 kDa in the strain UA159 Δ*1558c* (Supplementary Figure S2). Then, we conducted a comparative proteomic analysis between UA159 and UA159 Δ*1558c*. As shown in the volcano plot, there are 166 differentially expressed proteins (fold change > 1.2) between UA159 and UA159 Δ*1558c* (Figure 4A). Of these, 96 proteins were significantly upregulated, and 70 proteins were significantly downregulated (Figures 4A,B; Supplementary Tables S2, S3) in UA159 Δ*1558c*. Among the upregulated proteins, ClpL (encoded by *smu_956*) was the most significantly upregulated, showing high abundance (Figure 4A; Supplementary Figure S3; Supplementary Table S4).

Further analysis of the differentially expressed proteins (DEPs) revealed a diverse range of functional categories affected by the deletion of *smu_1558c*. These categories mainly include carbohydrate transport and metabolism, translation, ribosomal structure and biogenesis, amino acid transport and metabolism, and proteins with unknown functions (Figure 4B). To better understand the functional implications of DEPs in UA159 Δ*1558c*, we conducted enrichment analysis using the Gene Ontology (GO) and Kyoto Encyclopedia of Genes and Genomes (KEGG). As shown in Figure 4C, GO enrichment analysis unveiled the upregulated proteins were primarily enriched in transcription, DNA-templated, and PTS system, and the downregulated proteins were primarily enriched in peroxiredoxin activity. KEGG pathway analysis revealed that upregulated proteins were enriched in fructose and mannose metabolism and PTS systems, and the downregulated proteins were enriched in ABC transport systems (Figure 4D).

**Figure 5:**
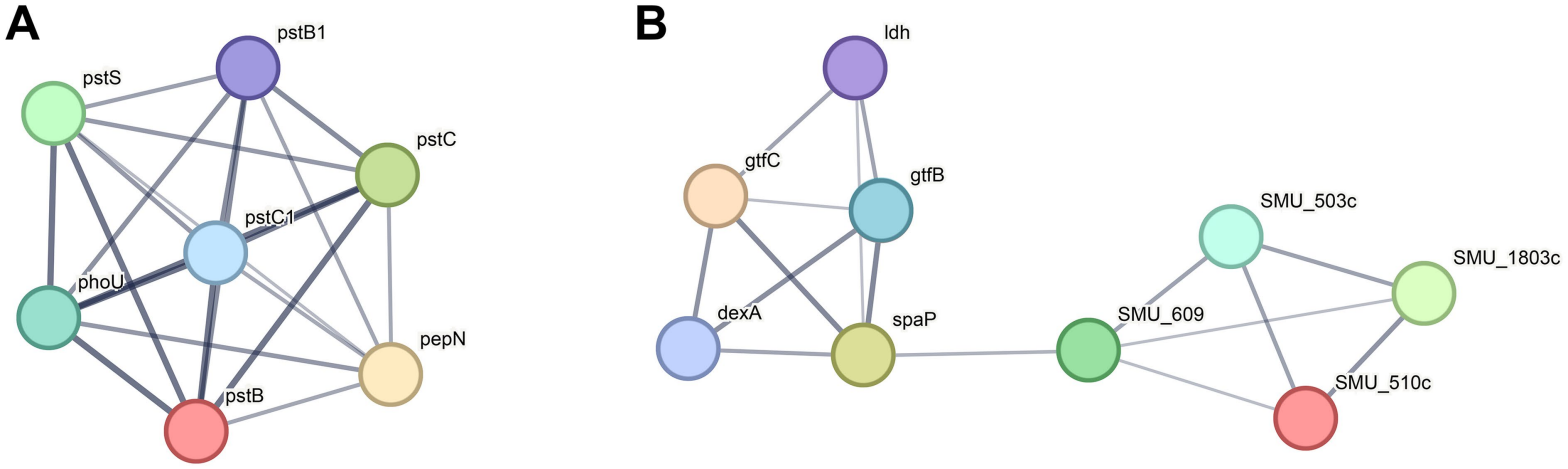
Top two clusters of highly interconnected downregulated proteins in UA159 Δ*1558c*. Interaction networks of proteins (listed by gene names) were analyzed using the MCODE plug-in toolkit in the Cytoscape software (version 3.9.1). **(A)** Cluster 1, enriched in ABC transporters. MCODE score = 7, nodes = 7, edges = 21. **(B)** Cluster 2, enriched in starch and sucrose metabolism. MCODE score = 4, nodes = 9, edges = 16. The detailed cluster information is in Supplementary Table S5.

To further understand the functional interactions of the DEPs in UA159 Δ*1558c*, we utilized the search tool for the retrieval of interacting genes/proteins (STRING) database to analyze protein–protein interaction (PPI) networks. Combining cluster analysis by the molecular complex detection (MCODE) module in Cytoscape v3.9.1, we characterized several highly interconnected networks (Figure 5; Supplementary Table S5). Notably, proteins in cluster 1 are predominantly associated with ABC transporters, while proteins in cluster 2 are mainly involved in starch and sucrose metabolism (Figure 5; Supplementary Table S5). In addition, it is noteworthy that both glucosyltransferase GtfB and GtfC, which are related to biofilm formation, were downregulated in cluster 2. This analysis aligns with the functional categories analysis, which indicated significant enrichment in pathways related to metabolism and transport.

These comprehensive analyses provide valuable insights into the regulatory mechanisms affected by *smu_1558c* deletion and highlight potential carbohydrate transport and metabolism pathways that may impact the growth and biofilm formation of *S. mutans*.

## 4 Discussion

Biofilm formation is a crucial virulence factor for *S. mutans*, contributing significantly to dental caries through its complex regulatory mechanisms involving various genetic and environmental factors (Shokeen et al., 2021). Previous studies have highlighted the roles of two-component systems, transcription factors, and sRNAs in regulating biofilm formation in *S. mutans* (Chen et al., 2021; Lei et al., 2019; Li et al., 2018; Yin et al., 2020). Acetyltransferase, responsible for the protein acetylation, also plays an essential role in bacterial biofilm formation (Armalytė et al., 2023; Lin et al., 2020). In this study, we focused on the GNAT family acetyltransferase SMU_1558c and its impact on *S. mutans* growth and biofilm formation.

The results showed that the deletion strain UA159 Δ*1558c* exhibited a pronounced growth defect during the logarithmic phase compared to the wild-type strain UA159. This growth impairment could be linked to disruptions in carbohydrate transport and metabolism, as revealed by our proteomic analysis. The downregulation of ABC transport systems and key metabolic pathways, such as fructose and mannose metabolism, suggests that *smu_1558c* may be involved in regulating nutrient acquisition and utilization (Luz et al., 2012; Davidson and Chen, 2004; Soni et al., 2020; Lee et al., 2018). Interestingly, the overexpression strain UA159/pDL278-*1558c* also showed impaired growth, although the effects were less pronounced than those observed in the deletion strain. This suggests that a balanced expression of *smu_1558c* is required for optimal growth and that both overexpression and deletion can disrupt these processes. This finding underscores the complexity of acetyltransferase-mediated regulation in *S. mutans*, where precise control of acetyltransferase expression is necessary for maintaining cellular homeostasis.

Dental biofilms provide a protective niche for *S. mutans* and enable them to persist in the oral cavity, which is fundamental to the cariogenicity of *S. mutans*. Our results showed that *smu_1558c* deletion significantly reduced biofilm formation and water-insoluble EPS production in both 6-h and 12-h biofilms. This is supported by the downregulation of glucosyltransferases GtfB and GtfC, key enzymes involved in synthesizing EPS that form the structural backbone of the biofilm matrix (Atta et al., 2024; Hoshino and Fujiwara, 2022; Tamesada et al., 2004). In addition, the significant reduction in colony-forming units (CFUs) in the *smu_1558c* deletion strain suggests that the diminished biofilm biomass and EPS production are partially due to lower bacterial cell numbers. The biofilm analysis using scanning electron microscopy (SEM) and confocal laser scanning microscopy (CLSM) revealed that the deletion of *smu_1558c* leads to a porous and less compact biofilm structure, consistent with the reduced biofilm biomass and EPS synthesis observed in the quantitative assays.

Proteomic analysis revealed extensive changes in protein expression profiles in the *smu_1558c* deletion strain, providing deeper insights into the underlying regulatory mechanisms. Among the 166 differentially expressed proteins, the most notable was ClpL, a Clp-like ATP-dependent protease, which was significantly upregulated in the deletion strain. ClpL is a member of the heat shock protein (Hsp) 100 family, which is unique to Gram-positive bacteria and plays a role in protein homeostasis under stress conditions (Park et al., 2015; Suokko et al., 2005). Interestingly, previous studies have shown that the deletion of *clpL* enhances biofilm formation in the presence of sucrose in *Staphylococcus aureus*, *Pseudomonas aeruginosa*, *Enterococcus faecalis*, and *Porphyromonas gingivalis* (Feng et al., 2021; Frees et al., 2004; He et al., 2019; Ju et al., 2021; Mawla et al., 2021; Zhang et al., 2015; Zheng et al., 2020). This suggests that the upregulation of ClpL may represent a compensatory mechanism in response to protein homeostasis disturbances caused by the loss of *smu_1558c*.

To fully elucidate the role of SMU_1558c in *S. mutans*, future studies should focus on acetylome profiling to identify specific acetylation targets of SMU_1558c and their regulatory effects (Liu et al., 2014; Sun et al., 2019). Understanding the precise mechanisms by which SMU_1558c modulates protein expression through acetylation will provide deeper insights into the regulatory networks governing bacterial physiology and pathogenicity.

In conclusion, our study highlights the significant role of the GNAT family acetyltransferase SMU_1558c in regulating the growth and biofilm formation of *S. mutans*. The deletion of *smu_1558c* leads to growth delay, reduced biofilm formation, and decreased EPS synthesis, primarily through the alterations in carbohydrate transport and metabolism pathways, as well as stress response mechanisms. These findings contribute to our understanding of the regulatory mechanisms underlying *S. mutans* biofilm formation and offer potential therapeutic strategies for disrupting biofilm formation and preventing dental caries.

## Data Availability

The original contributions presented in the study are included in the article/Supplementary material, further inquiries can be directed to the corresponding authors.

## Footnotes

^1^ http://www.majorbio.com

^2^ http://david.abcc.ncifcrf.gov/

^3^ http://string.embl.de/

## Supplementary material

SUPPLEMENTARY FIGURE S1 Growth curves of the GNAT family acetyltransferases gene deficient strains. The growth of UA159 and its derivative mutants (UA159 Δ*386*::IFDC2, UA159 Δ*639*::IFDC2, UA159 Δ*844*::IFDC2, UA159 Δ*850*::IFDC2, UA159 Δ*1072c*::IFDC2, UA159 Δ*1154c*::IFDC2, UA159 Δ*1253c*::IFDC2, UA159 Δ*1392c*::IFDC2, UA159 Δ*1483c*::IFDC2, UA159 Δ*1558c*::IFDC2, UA159 Δ*1654c*::IFDC2, UA159 Δ*1730c*::IFDC2, UA159 Δ*2055*::IFDC2, and UA159 Δ*2072c*::IFDC2) was monitored in anaerobic condition for 12 h by measuring OD_600 nm_ at regular intervals.

SUPPLEMENTARY FIGURE S2 SDS-PAGE analysis of total protein extracts from UA159 and its derivative strains. SDS-PAGE gel showed the total protein profiles of wild-type UA159, UA159/pDL278, UA159 Δ*1558c*, and UA159/pDL278-*1558c*. A prominent upregulated protein band in UA159 Δ*1558c* in the molecular weight range of 70 kDa to 100 kDa.

SUPPLEMENTARY FIGURE S3 Protein-protein interaction (PPI) network of differentially expressed proteins in UA159 Δ*1558c*. The network illustrates interactions between upregulated and downregulated proteins in Δ*1558c*, based on fold changes from proteomic analysis. Each node represents a protein, with green indicating downregulated proteins and red indicating upregulated proteins. The intensity of the color corresponds to the degree of fold change: dark red indicates upregulation by 2-fold, red by 1.5-fold, and light red by 1.2-fold; dark green indicates downregulation by 2-fold, green by 1.5-fold, and light green by 1.2-fold. The interaction data was obtained from the STRING database, and the network was analyzed using CYTOSCAPE V3.9.1. Notably, ClpL is significantly upregulated, as highlighted in dark red.

## References

[ref1] Ajdic D. McShan W. M. McLaughlin R. E. Savic G. Chang J. Carson M. B.. (2002). Genome sequence of *Streptococcus mutans* UA159, a cariogenic dental pathogen. Proc. Natl. Acad. Sci. USA 99, 14434–14439. doi: 10.1073/pnas.172501299, PMID: 12397186 PMC137901

[ref2] Armalytė J. Čepauskas A. Šakalytė G. Martinkus J. Skerniškytė J. Martens C.. (2023). A polyamine acetyltransferase regulates the motility and biofilm formation of *Acinetobacter baumannii*. Nat. Commun. 14:3531. doi: 10.1038/s41467-023-39316-5, PMID: 37316480 PMC10267138

[ref3] Atta L. Mushtaq M. Siddiqui A. R. Khalid A. Ul-Haq Z. (2024). Targeting glucosyltransferases to combat dental caries: current perspectives and future prospects. Int. J. Biol. Macromol. 278:134645. doi: 10.1016/j.ijbiomac.2024.134645, PMID: 39128764

[ref4] Burckhardt R. M. Escalante-Semerena J. C. (2020). Small-molecule acetylation by GCN5-related N-acetyltransferases in Bacteria. Microbiol. Mol. Biol. Rev. 84:e00090-19. doi: 10.1128/MMBR.00090-19, PMID: 32295819 PMC7160885

[ref5] Carabetta V. J. Cristea I. M. (2017). Regulation, function, and detection of protein acetylation in Bacteria. J. Bacteriol. 199:e00107-17. doi: 10.1128/JB.00107-17, PMID: 28439035 PMC5527388

[ref6] Chen J. Zhang A. Xiang Z. Lu M. Huang P. Gong T.. (2021). EpsR negatively regulates *Streptococcus mutans* exopolysaccharide synthesis. J. Dent. Res. 100, 968–976. doi: 10.1177/00220345211000668, PMID: 33749354

[ref7] Christensen D. G. Baumgartner J. T. Xie X. Jew K. M. Basisty N. Schilling B.. (2019). Mechanisms, detection, and relevance of protein acetylation in prokaryotes. MBio 10:e02708-18. doi: 10.1128/mBio.02708-18 30967470 PMC6456759

[ref8] Cugini C. Shanmugam M. Landge N. Ramasubbu N. (2019). The role of exopolysaccharides in Oral biofilms. J. Dent. Res. 98, 739–745. doi: 10.1177/0022034519845001, PMID: 31009580 PMC6589894

[ref9] Davidson A. L. Chen J. (2004). ATP-binding cassette transporters in bacteria. Annu. Rev. Biochem. 73, 241–268. doi: 10.1146/annurev.biochem.73.011303.073626, PMID: 15189142

[ref10] Feng Y. Wang H. Lu H. E. Yi L. Hong L. I. (2021). Effects of ClpP protease on biofilm formation of *Enterococcus faecalis*. J. Appl. Oral Sci. 29:e20200733. doi: 10.1590/1678-7757-2020-0733, PMID: 33656065 PMC7934281

[ref11] Frees D. Chastanet A. Qazi S. Sørensen K. Hill P. Msadek T.. (2004). Clp ATPases are required for stress tolerance, intracellular replication and biofilm formation in *Staphylococcus aureus*. Mol. Microbiol. 54, 1445–1462. doi: 10.1111/j.1365-2958.2004.04368.x, PMID: 15554981

[ref12] Gong T. He X. Chen J. Tang B. Zheng T. Jing M.. (2021). Transcriptional profiling reveals the importance of RcrR in the regulation of multiple sugar transportation and biofilm formation in *Streptococcus mutans*. mSystems 6:e0078821. doi: 10.1128/mSystems.00788-21, PMID: 34427509 PMC8407328

[ref13] He L. Wang H. Zhang R. Li H. (2019). The regulation of *Porphyromonas gingivalis* biofilm formation by ClpP. Biochem. Biophys. Res. Commun. 509, 335–340. doi: 10.1016/j.bbrc.2018.12.071, PMID: 30579592

[ref14] Hoshino T. Fujiwara T. (2022). The findings of glucosyltransferase enzymes derived from oral streptococci. Jpn. Dent. Sci. Rev. 58, 328–335. doi: 10.1016/j.jdsr.2022.10.003, PMID: 36340584 PMC9630777

[ref15] Ju Y. An Q. Zhang Y. Sun K. Bai L. Luo Y. (2021). Recent advances in Clp protease modulation to address virulence, resistance and persistence of MRSA infection. Drug Discov. Today 26, 2190–2197. doi: 10.1016/j.drudis.2021.05.014, PMID: 34048895

[ref16] Klein M. I. Hwang G. Santos P. H. Campanella O. H. Koo H. (2015). *Streptococcus mutans*-derived extracellular matrix in cariogenic oral biofilms. Front. Cell. Infect. Microbiol. 5:10. doi: 10.3389/fcimb.2015.00010, PMID: 25763359 PMC4327733

[ref17] Koo H. Falsetta M. L. Klein M. I. (2013). The exopolysaccharide matrix: a virulence determinant of cariogenic biofilm. J. Dent. Res. 92, 1065–1073. doi: 10.1177/0022034513504218, PMID: 24045647 PMC3834652

[ref18] Lamont R. J. Koo H. Hajishengallis G. (2018). The oral microbiota: dynamic communities and host interactions. Nat. Rev. Microbiol. 16, 745–759. doi: 10.1038/s41579-018-0089-x, PMID: 30301974 PMC6278837

[ref7001] LeBlanc D. J. Lee L. N. Abu-Al-Jaibat A. (1992). Molecular, genetic, and functional analysis of the basic replicon of pVA380-1, a plasmid of oral streptococcal origin. Plasmid. 28, 130–145. doi: 10.1016/0147-619x(92)90044-b 1409970

[ref19] Lee Y. Song S. Sheng L. Zhu L. Kim J. S. Wood T. K. (2018). Substrate binding protein DppA1 of ABC transporter DppBCDF increases biofilm formation in *Pseudomonas aeruginosa* by inhibiting Pf5 prophage lysis. Front. Microbiol. 9:30. doi: 10.3389/fmicb.2018.00030, PMID: 29416528 PMC5787571

[ref20] Lei L. Long L. Yang X. Qiu Y. Zeng Y. Hu T.. (2019). The VicRK two-component system regulates *Streptococcus mutans* virulence. Curr. Issues Mol. Biol. 32, 167–200. doi: 10.21775/cimb.032.167, PMID: 31166172

[ref21] Li Z. Xiang Z. Zeng J. Li Y. Li J. (2018). A GntR family transcription factor in *Streptococcus mutans* regulates biofilm formation and expression of multiple sugar transporter genes. Front. Microbiol. 9:3224. doi: 10.3389/fmicb.2018.03224, PMID: 30692967 PMC6340165

[ref22] Lin C. J. Hou Y. H. Chen Y. L. (2020). The histone acetyltransferase GcnE regulates conidiation and biofilm formation in *Aspergillus fumigatus*. Med. Mycol. 58, 248–259. doi: 10.1093/mmy/myz043, PMID: 31100153

[ref23] Liu M. Guo L. Fu Y. Huo M. Qi Q. Zhao G. (2021). Bacterial protein acetylation and its role in cellular physiology and metabolic regulation. Biotechnol. Adv. 53:107842. doi: 10.1016/j.biotechadv.2021.107842, PMID: 34624455

[ref24] Liu F. Yang M. Wang X. Yang S. Gu J. Zhou J.. (2014). Acetylome analysis reveals diverse functions of lysine acetylation in *Mycobacterium tuberculosis*. Mol. Cell. Proteomics 13, 3352–3366. doi: 10.1074/mcp.M114.041962, PMID: 25180227 PMC4256489

[ref25] Luz D. E. Nepomuceno R. S. Spira B. Ferreira R. C. (2012). The Pst system of *Streptococcus mutans* is important for phosphate transport and adhesion to abiotic surfaces. Mol Oral Microbiol 27, 172–181. doi: 10.1111/j.2041-1014.2012.00641.x, PMID: 22520387

[ref26] Ma Q. Pan Y. Chen Y. Yu S. Huang J. Liu Y.. (2022). Acetylation of lactate dehydrogenase negatively regulates the Acidogenicity of *Streptococcus mutans*. MBio 13:e0201322. doi: 10.1128/mbio.02013-22, PMID: 36043788 PMC9600946

[ref27] Ma Q. Pan Y. Chen Y. Yu S. Huang J. Liu Y.. (2021). Acetylation of glucosyltransferases regulates *Streptococcus mutans* biofilm formation and virulence. PLoS Pathog. 17:e1010134. doi: 10.1371/journal.ppat.1010134, PMID: 34860858 PMC8673623

[ref28] Ma Q. Zhang Q. Chen Y. Yu S. Huang J. Liu Y.. (2021). Post-translational modifications in Oral Bacteria and their functional impact. Front. Microbiol. 12:784923. doi: 10.3389/fmicb.2021.784923, PMID: 34925293 PMC8674579

[ref29] Mawla G. D. Hall B. M. Cárcamo-Oyarce G. Grant R. A. Zhang J. J. Kardon J. R.. (2021). ClpP1P2 peptidase activity promotes biofilm formation in *Pseudomonas aeruginosa*. Mol. Microbiol. 115, 1094–1109. doi: 10.1111/mmi.14649, PMID: 33231899 PMC8141546

[ref30] Narita T. Weinert B. T. Choudhary C. (2019). Functions and mechanisms of non-histone protein acetylation. Nat. Rev. Mol. Cell Biol. 20, 156–174. doi: 10.1038/s41580-018-0081-3, PMID: 30467427

[ref31] Nishimura J. Saito T. Yoneyama H. Bai L. Okumura K. Isogai E. (2012). Biofilm formation by Streptococcus mutans and related Bacteria. Adv. Microbiol. 2, 208–215. doi: 10.4236/aim.2012.23025

[ref32] Park S. S. Kwon H. Y. Tran T. D. Choi M. H. Jung S. H. Lee S.. (2015). ClpL is a chaperone without auxiliary factors. FEBS J. 282, 1352–1367. doi: 10.1111/febs.13228, PMID: 25662392

[ref33] Pitts N. B. Zero D. T. Marsh P. D. Ekstrand K. Weintraub J. A. Ramos-Gomez F.. (2017). Dental caries. Nat. Rev. Dis. Primers 3:17030. doi: 10.1038/nrdp.2017.30 28540937

[ref34] Ren J. Sang Y. Lu J. Yao Y. F. (2017). Protein acetylation and its role in bacterial virulence. Trends Microbiol. 25, 768–779. doi: 10.1016/j.tim.2017.04.001, PMID: 28462789

[ref35] Rosier B. T. Marsh P. D. Mira A. (2018). Resilience of the Oral microbiota in health: mechanisms that prevent Dysbiosis. J. Dent. Res. 97, 371–380. doi: 10.1177/0022034517742139, PMID: 29195050

[ref36] Shokeen B. Dinis M. D. B. Haghighi F. Tran N. C. Lux R. (2021). Omics and interspecies interaction. Periodontol. 2000 85, 101–111. doi: 10.1111/prd.12354, PMID: 33226675

[ref37] Shvedunova M. Akhtar A. (2022). Modulation of cellular processes by histone and non-histone protein acetylation. Nat. Rev. Mol. Cell Biol. 23, 329–349. doi: 10.1038/s41580-021-00441-y, PMID: 35042977

[ref38] Simón-Soro A. Mira A. (2015). Solving the etiology of dental caries. Trends Microbiol. 23, 76–82. doi: 10.1016/j.tim.2014.10.010, PMID: 25435135

[ref39] Soni D. K. Dubey S. K. Bhatnagar R. (2020). ATP-binding cassette (ABC) import systems of *Mycobacterium tuberculosis*: target for drug and vaccine development. Emerg. Microbes Infect. 9, 207–220. doi: 10.1080/22221751.2020.1714488, PMID: 31985348 PMC7034087

[ref40] Sun L. Yao Z. Guo Z. Zhang L. Wang Y. Mao R.. (2019). Comprehensive analysis of the lysine acetylome in *Aeromonas hydrophila* reveals cross-talk between lysine acetylation and succinylation in LuxS. Emerg. Microbes Infect. 8, 1229–1239. doi: 10.1080/22221751.2019.1656549, PMID: 31448697 PMC6735345

[ref41] Suokko A. Savijoki K. Malinen E. Palva A. Varmanen P. (2005). Characterization of a mobile clpL gene from *Lactobacillus rhamnosus*. Appl. Environ. Microbiol. 71, 2061–2069. doi: 10.1128/AEM.71.4.2061-2069.2005, PMID: 15812039 PMC1082546

[ref42] Tamesada M. Kawabata S. Fujiwara T. Hamada S. (2004). Synergistic effects of streptococcal glucosyltransferases on adhesive biofilm formation. J. Dent. Res. 83, 874–879. doi: 10.1177/154405910408301110, PMID: 15505239

[ref43] Tian Y. He X. Torralba M. Yooseph S. Nelson K. E. Lux R.. (2010). Using DGGE profiling to develop a novel culture medium suitable for oral microbial communities. Mol Oral Microbiol 25, 357–367. doi: 10.1111/j.2041-1014.2010.00585.x, PMID: 20883224 PMC2951289

[ref44] VanDrisse C. M. Escalante-Semerena J. C. (2019). Protein acetylation in Bacteria. Ann. Rev. Microbiol. 73, 111–132. doi: 10.1146/annurev-micro-020518-115526, PMID: 31091420 PMC6736716

[ref45] Xiang Z. Li Z. Ren Z. Zeng J. Peng X. Li Y.. (2019). EzrA, a cell shape regulator contributing to biofilm formation and competitiveness in *Streptococcus mutans*. Mol Oral Microbiol 34, 194–208. doi: 10.1111/omi.12264, PMID: 31287946

[ref46] Xiao J. Klein M. I. Falsetta M. L. Lu B. Delahunty C. M. Yates J. R. 3rd. (2012). The exopolysaccharide matrix modulates the interaction between 3D architecture and virulence of a mixed-species oral biofilm. PLoS Pathog. 8:e1002623. doi: 10.1371/journal.ppat.1002623, PMID: 22496649 PMC3320608

[ref47] Xie Z. Okinaga T. Qi F. Zhang Z. Merritt J. (2011). Cloning-independent and counterselectable markerless mutagenesis system in *Streptococcus mutans*. Appl. Environ. Microbiol. 77, 8025–8033. doi: 10.1128/AEM.06362-11, PMID: 21948849 PMC3208986

[ref48] Xu X. Zhou X. D. Wu C. D. (2011). The tea catechin epigallocatechin gallate suppresses cariogenic virulence factors of *Streptococcus mutans*. Antimicrob. Agents Chemother. 55, 1229–1236. doi: 10.1128/AAC.01016-10, PMID: 21149622 PMC3067078

[ref49] Yin L. Zhu W. Chen D. Zhou Y. Lin H. (2020). Small noncoding RNA sRNA0426 is involved in regulating biofilm formation in *Streptococcus mutans*. Microbiology 9:e1096. doi: 10.1002/mbo3.1096, PMID: 32633012 PMC7521000

[ref50] Yu S. Ma Q. Huang J. Liu Y. Li J. Wang Y.. (2024). SMU_1361c regulates the oxidative stress response of *Streptococcus mutans*. Appl. Environ. Microbiol. 90:e0187123. doi: 10.1128/aem.01871-23, PMID: 38299814 PMC10880606

[ref51] Zhang J. Q. Hou X. H. Song X. Y. Ma X. B. Zhao Y. X. Zhang S. Y. (2015). ClpP affects biofilm formation of *Streptococcus mutans* differently in the presence of cariogenic carbohydrates through regulating gtfBC and ftf. Curr. Microbiol. 70, 716–723. doi: 10.1007/s00284-015-0779-9, PMID: 25645737

[ref52] Zhang Q. Ma Q. Wang Y. Wu H. Zou J. (2021). Molecular mechanisms of inhibiting glucosyltransferases for biofilm formation in *Streptococcus mutans*. Int. J. Oral Sci. 13:30. doi: 10.1038/s41368-021-00137-1, PMID: 34588414 PMC8481554

[ref53] Zheng J. Wu Y. Lin Z. Wang G. Jiang S. Sun X.. (2020). ClpP participates in stress tolerance, biofilm formation, antimicrobial tolerance, and virulence of *Enterococcus faecalis*. BMC Microbiol. 20:30. doi: 10.1186/s12866-020-1719-9, PMID: 32033530 PMC7006429

