# Supplementary material for: smu_1558c-mediated regulation of growth and biofilm formation in Streptococcus mutans
Source: Front Microbiol. 2025 Jan 17;15:1507928. doi: 10.3389/fmicb.2024.1507928 (PMC11782273; doi:10.3389/fmicb.2024.1507928)
Supplement: SUPPLEMENTARY FIGURE S1 — Growth curves of the GNAT family acetyltransferases gene deficient strains. The growth of UA159 and its derivative mutants (UA159 Δ386::IFDC2, UA159 Δ639::IFDC2, UA159 Δ844::IFDC2, UA159 Δ850::IFDC2, UA159 Δ1072c::IFDC2, UA159 Δ1154c::IFDC2, UA159 Δ1253c::IFDC2, UA159 Δ1392c::IFDC2, UA159 Δ1483c::IFDC2, UA159 Δ1558c::IFDC2, UA159 Δ1654c::IFDC2, UA159 Δ1730c::IFDC2, UA159 Δ2055::IFDC2, and UA159 Δ2072c::IFDC2) was monitored in anaerobic condition for 12 h by measuring OD600 nm at regular intervals. [file Supplementary_file_1.zip › Supplementary Figures and Figure Legends.pdf]

Supplementary Figures and Figure Legends:

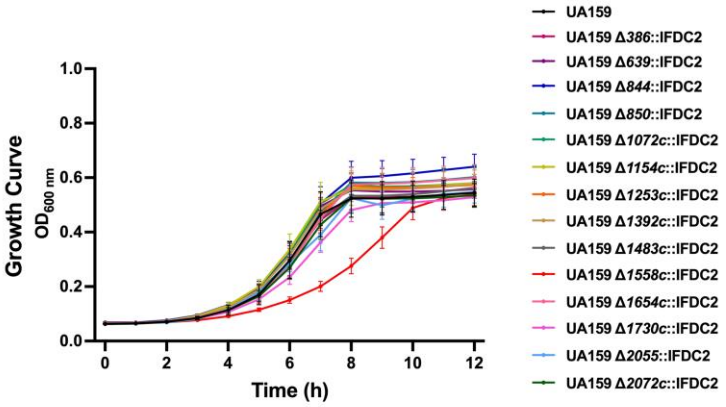

Fig. S1. Growth curves of the GNAT family acetyltransferases gene deficient strains. The growth of UA159 and its derivative mutants (UA159 Δ*386*::IFDC2, UA159 Δ*639*::IFDC2, UA159 Δ*844*::IFDC2, UA159 Δ*850*::IFDC2, UA159 Δ*1072c*::IFDC2, UA159 Δ*1154c*::IFDC2, UA159 Δ*1253c*::IFDC2, UA159 Δ*1392c*::IFDC2, UA159 Δ*1483c*::IFDC2, UA159 Δ*1558c*::IFDC2, UA159 Δ*1654c*::IFDC2, UA159 Δ*1730c*::IFDC2, UA159 Δ*2055*::IFDC2, and UA159 Δ*2072c*::IFDC2) was monitored in anaerobic condition for 12 h by measuring $OD_{600\ nm}$ at regular intervals.

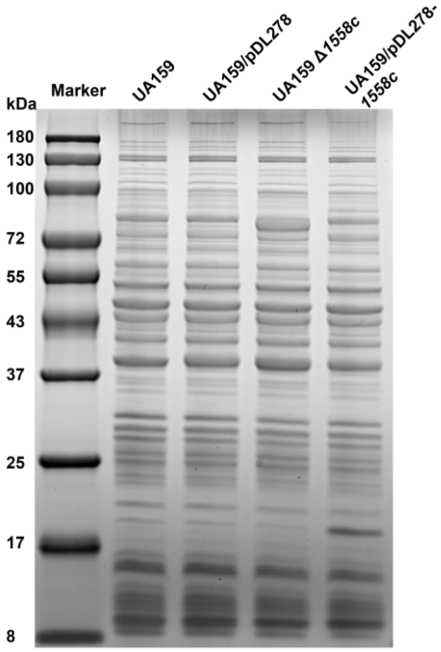

Fig. S2. SDS-PAGE analysis of total protein extracts from UA159 and its derivative strains. SDS-PAGE gel showed the total protein profiles of wild-type UA159, UA159/pDL278, UA159 Δ*1558c*, and UA159/pDL278-*1558c*. A prominent upregulated protein band in UA159 Δ*1558c* in the molecular weight range of 70 kDa to 100 kDa.

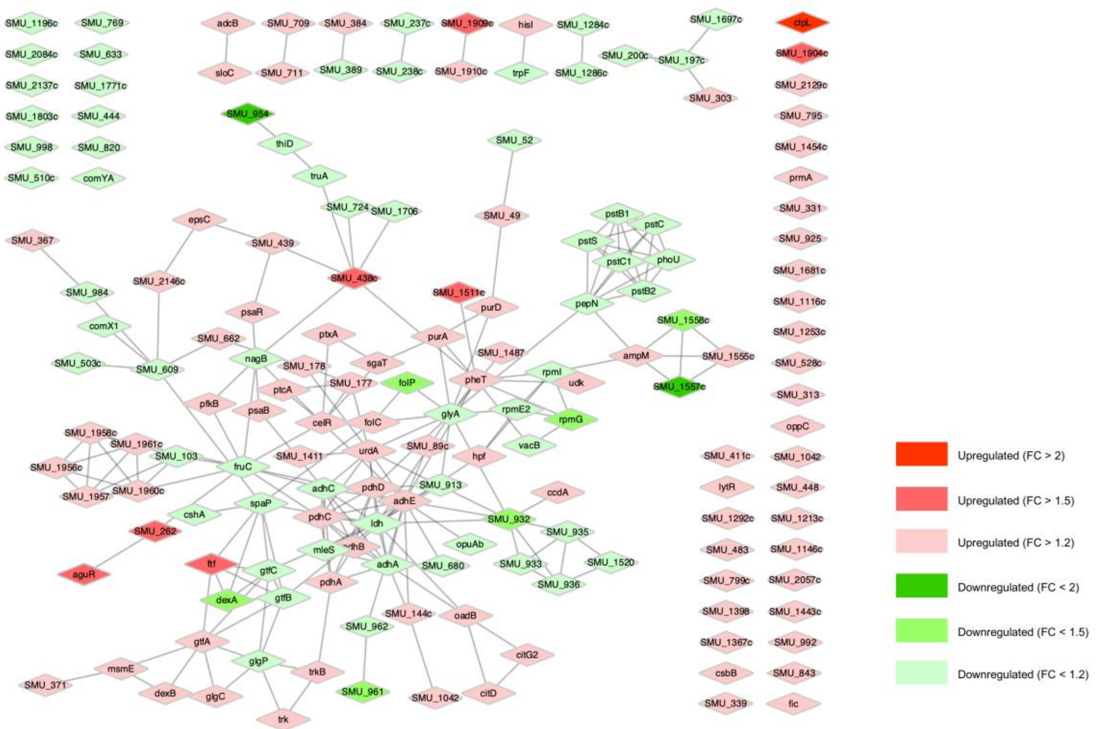

Fig. S3. Protein-protein interaction (PPI) network of differentially expressed proteins in UA159 Δ*1558c*. The network illustrates interactions between upregulated and downregulated proteins in Δ*1558c*, based on fold changes from proteomic analysis. Each node represents a protein, with green indicating downregulated proteins and red indicating upregulated proteins. The intensity of the color corresponds to the degree of fold change: dark red indicates upregulation by 2-fold, red by 1.5-fold, and light red by 1.2-fold; dark green indicates downregulation by 2-fold, green by 1.5-fold, and light green by 1.2-fold. The interaction data was obtained from the STRING database, and the network was analyzed using CYTOSCAPE V3.9.1. Notably, ClpL is significantly upregulated, as highlighted in dark red.

Supplementary Tables (separate file)

Table S1. Bacterial strains, plasmids, and primers used in this study

Table S2. List of proteins expressed in UA159 Δ*1558c*

Table S3. Differentially expressed proteins greater than 1.2-fold in UA159 Δ*1558c*

Table S4. Protein-protein interaction networks of differentially expressed proteins between UA159 and UA159 Δ1558c

Table S5. Protein-protein interaction networks of downregulated proteins in UA159 Δ*1558c*
