# Supplementary figures and images for: smu_1558c-mediated regulation of growth and biofilm formation in Streptococcus mutans
Source: Front Microbiol. 2025 Jan 17;15:1507928. doi: 10.3389/fmicb.2024.1507928 (PMC11782273; doi:10.3389/fmicb.2024.1507928)

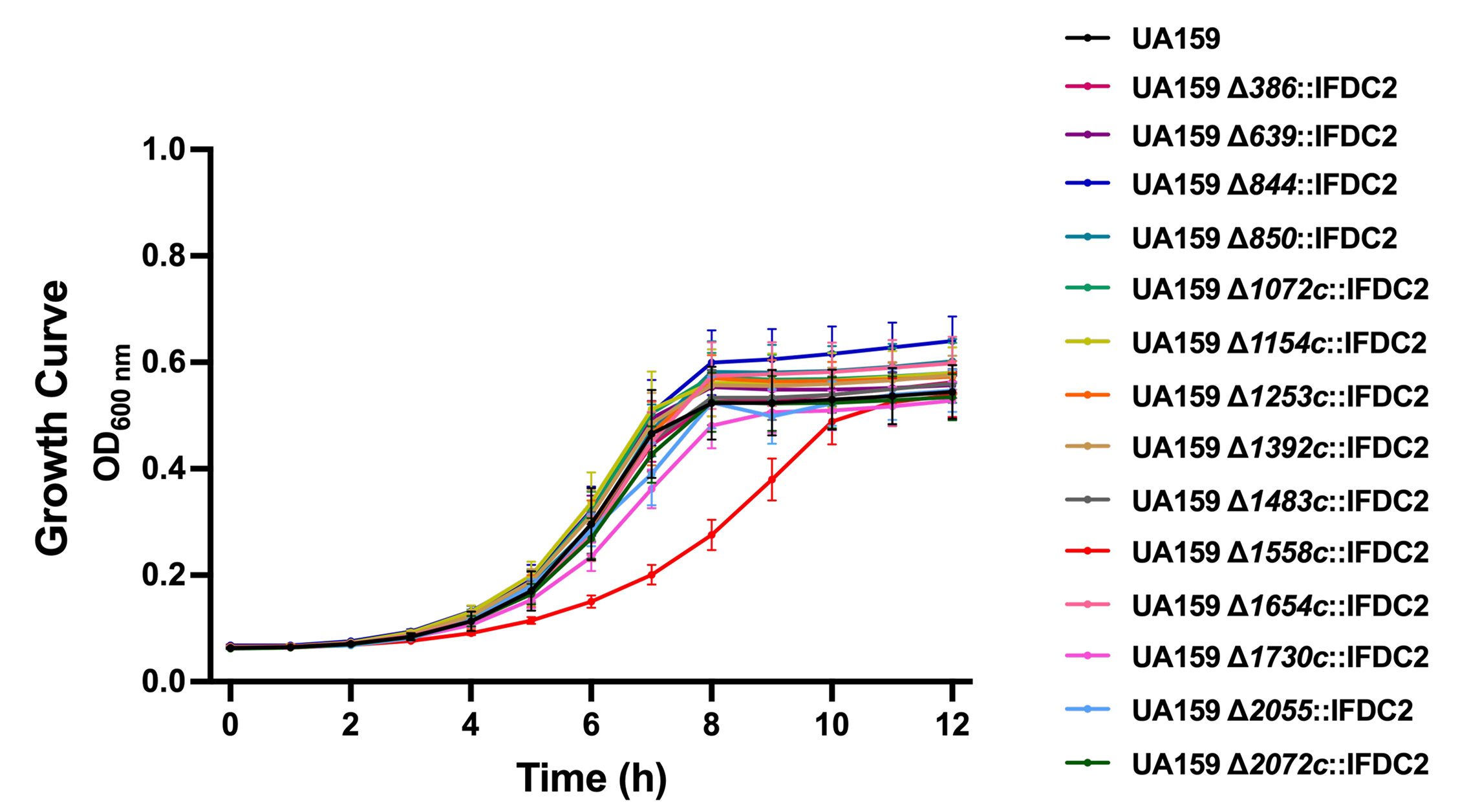

Supplement: SUPPLEMENTARY FIGURE S1 — Growth curves of the GNAT family acetyltransferases gene deficient strains. The growth of UA159 and its derivative mutants (UA159 Δ386::IFDC2, UA159 Δ639::IFDC2, UA159 Δ844::IFDC2, UA159 Δ850::IFDC2, UA159 Δ1072c::IFDC2, UA159 Δ1154c::IFDC2, UA159 Δ1253c::IFDC2, UA159 Δ1392c::IFDC2, UA159 Δ1483c::IFDC2, UA159 Δ1558c::IFDC2, UA159 Δ1654c::IFDC2, UA159 Δ1730c::IFDC2, UA159 Δ2055::IFDC2, and UA159 Δ2072c::IFDC2) was monitored in anaerobic condition for 12 h by measuring OD600 nm at regular intervals. [file Supplementary_file_1.zip › Fig. S1.tif]

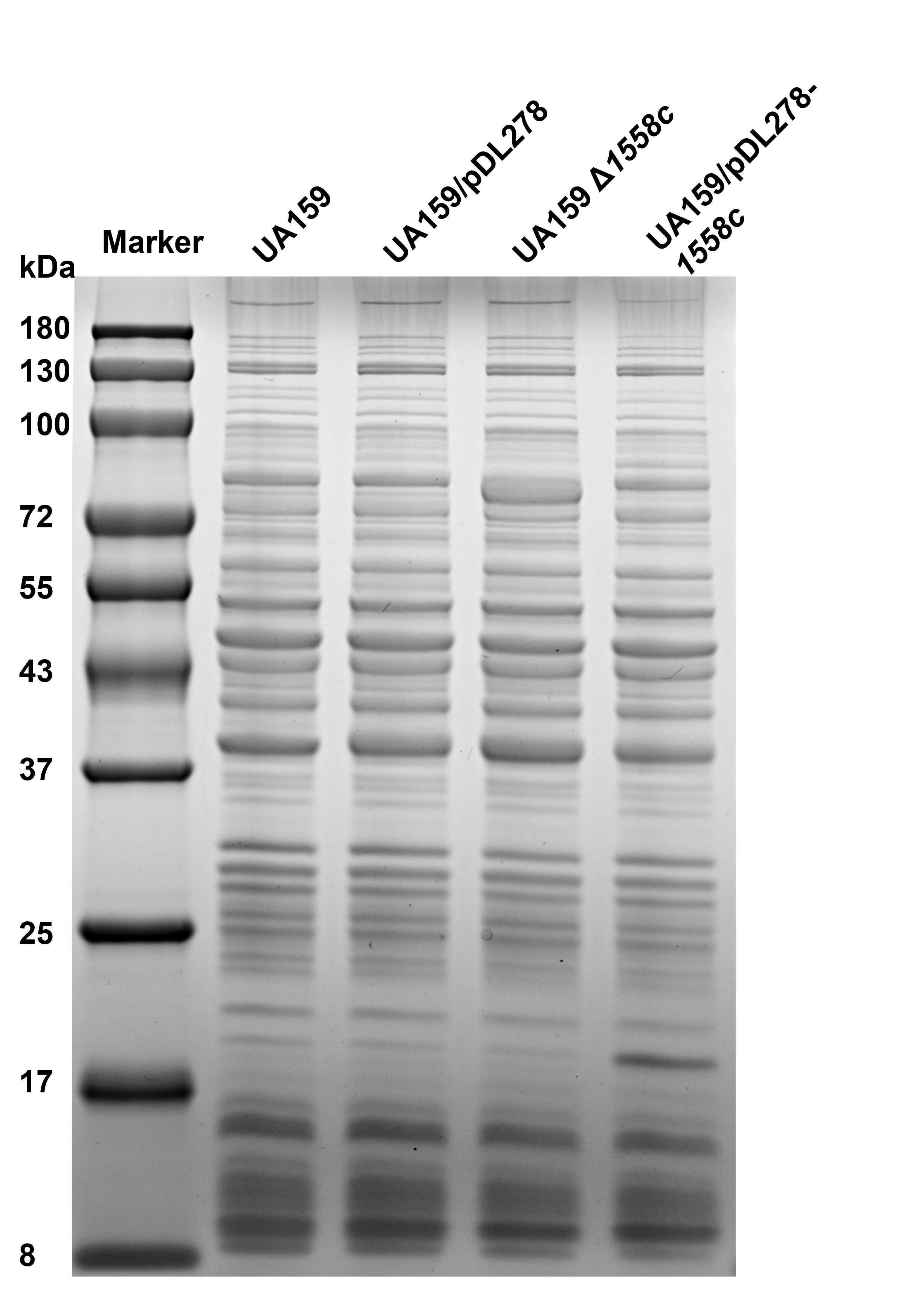

Supplement: SUPPLEMENTARY FIGURE S1 — Growth curves of the GNAT family acetyltransferases gene deficient strains. The growth of UA159 and its derivative mutants (UA159 Δ386::IFDC2, UA159 Δ639::IFDC2, UA159 Δ844::IFDC2, UA159 Δ850::IFDC2, UA159 Δ1072c::IFDC2, UA159 Δ1154c::IFDC2, UA159 Δ1253c::IFDC2, UA159 Δ1392c::IFDC2, UA159 Δ1483c::IFDC2, UA159 Δ1558c::IFDC2, UA159 Δ1654c::IFDC2, UA159 Δ1730c::IFDC2, UA159 Δ2055::IFDC2, and UA159 Δ2072c::IFDC2) was monitored in anaerobic condition for 12 h by measuring OD600 nm at regular intervals. [file Supplementary_file_1.zip › Fig. S2.tif]

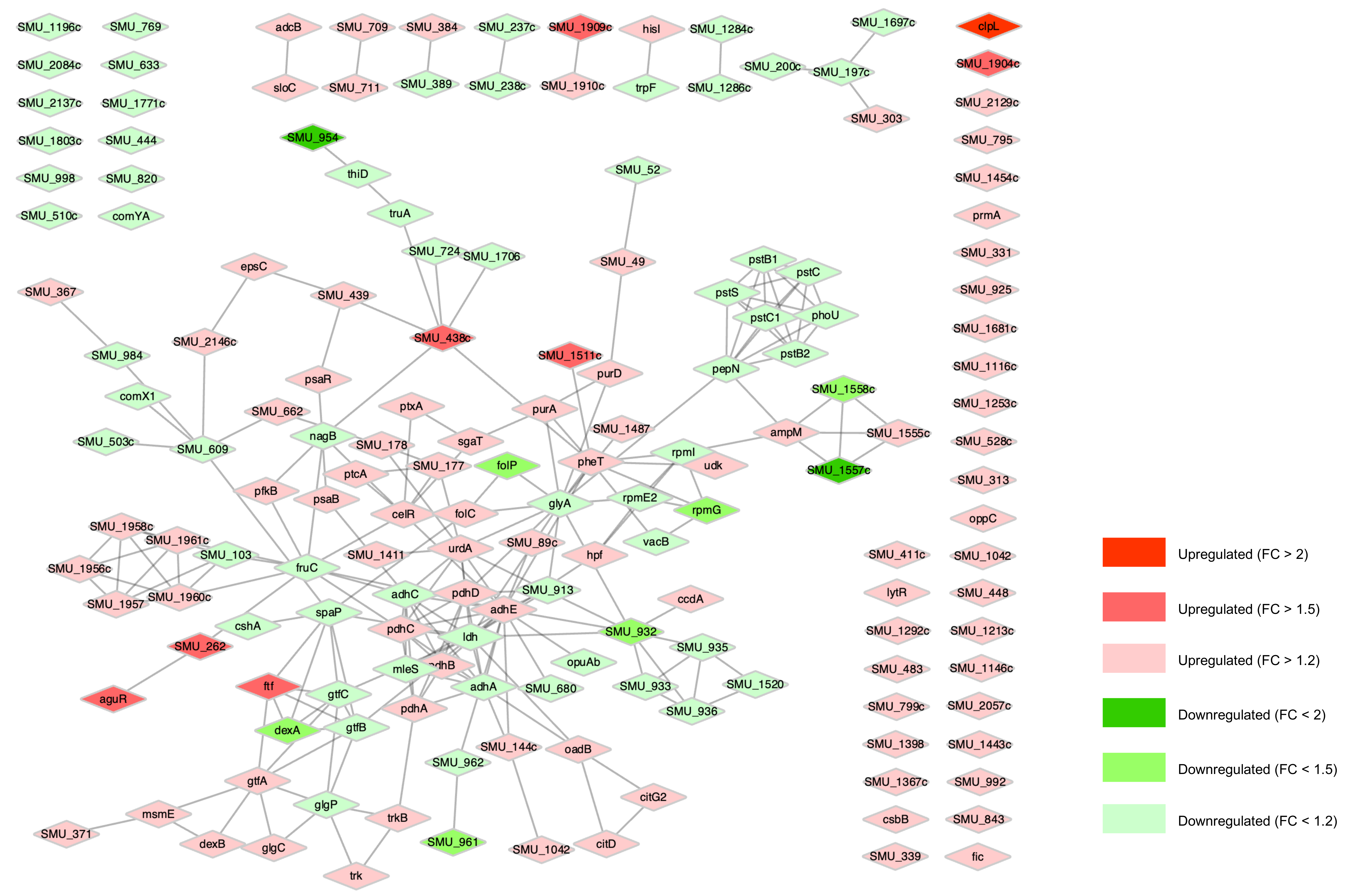

Supplement: SUPPLEMENTARY FIGURE S1 — Growth curves of the GNAT family acetyltransferases gene deficient strains. The growth of UA159 and its derivative mutants (UA159 Δ386::IFDC2, UA159 Δ639::IFDC2, UA159 Δ844::IFDC2, UA159 Δ850::IFDC2, UA159 Δ1072c::IFDC2, UA159 Δ1154c::IFDC2, UA159 Δ1253c::IFDC2, UA159 Δ1392c::IFDC2, UA159 Δ1483c::IFDC2, UA159 Δ1558c::IFDC2, UA159 Δ1654c::IFDC2, UA159 Δ1730c::IFDC2, UA159 Δ2055::IFDC2, and UA159 Δ2072c::IFDC2) was monitored in anaerobic condition for 12 h by measuring OD600 nm at regular intervals. [file Supplementary_file_1.zip › Fig. S3.tif]
